# Mediolateral damping of an overhead body weight support system assists stability during treadmill walking

**DOI:** 10.1186/s12984-020-00735-w

**Published:** 2020-08-10

**Authors:** M. Bannwart, S. L. Bayer, N. König Ignasiak, M. Bolliger, G. Rauter, C. A. Easthope

**Affiliations:** 1 Spinal Cord Injury Center, Balgrist University Hospital, University of Zurich, Zurich, Switzerland; 2 Sensory Motor Systems Laboratory, Department of Health Sciences and Technology, Swiss Federal Institute of Technology, Zurich, Switzerland; 3 Department of Physical Therapy, Chapman University, Irvine, USA; 4 BIROMED-Laboratory, Department of Biomedical Engineering, University of Basel, Basel, Switzerland; 5 cereneo Center for Interdisciplinary Research, Vitznau, Switzerland

**Keywords:** Body weight support, Mediolateral stability, Balance, Gait, Rehabilitation

## Abstract

**Background:**

Body weight support systems with three or more degrees of freedom (3-DoF) are permissive and safe environments that provide unloading and allow unrestricted movement in any direction. This enables training of walking and balance control at an early stage in rehabilitation. Transparent systems generate a support force vector that is near vertical at all positions in the workspace to only minimally interfere with natural movement patterns. Patients with impaired balance, however, may benefit from additional mediolateral support that can be adjusted according to their capacity. An elegant solution for providing balance support might be by rendering viscous damping along the mediolateral axis via the software controller. Before use with patients, we evaluated if control-rendered mediolateral damping evokes the desired stability enhancement in able-bodied individuals.

**Methods:**

A transparent, cable-driven robotic body weight support system (FLOAT) was used to provide transparent body weight support with and without mediolateral damping to 21 able-bodied volunteers while walking at preferred gait velocity on a treadmill. Stability metrics reflecting resistance to small and large perturbations were derived from walking kinematics and compared between conditions and to free walking.

**Results:**

Compared to free walking, the application of body weight support per-se resulted in gait alterations typically associated with body weight support, namely increased step length and swing phase. Frontal plane dynamic stability, measured by kinematic variability and nonlinear dynamics of the center of mass, was increased under body weight support, indicating reduced balance requirements in both damped and undamped support conditions. Adding damping to the body weight support resulted in a greater increase of frontal plane stability.

**Conclusion:**

Adding mediolateral damping to 3-DoF body weight support systems is an effective method of increasing frontal plane stability during walking in able-bodied participants. Building on these results, adjustable mediolateral damping could enable therapists to select combinations of unloading and stability specifically for each patient and to adapt this in a task specific manner. This could extend the impact of transparent 3-DoF body weight support systems, enabling training of gait and active balance from an early time point onwards in the rehabilitation process for a wide range of mobility activities of daily life.

## Background

In the past 3 decades, neurological injuries have been the leading cause of disease burden and second leading cause of deaths worldwide [1]. Around 60% of the affected patients manifest gait impairments [2], which contribute strongly to the disease burden. Locomotor rehabilitation programs aim at rehabilitating patients’ walking capacity and reducing gait impairments. Patients not able to safely bear full body weight struggle without large assistance and are prone to adopt compensatory movement strategies. Body weight support (BWS) is a promising path to retrain physiological walking and avoid the development of compensatory patterns. Testimony to this are the numerous devices on the market and currently in development and the rising adoption in rehabilitation taxonomy [3]. BWS is generally provided through a harness which applies vertical (VT) forces to the pelvis or trunk to achieve partial gravity reduction. Together with fall prevention mechanisms, this creates a safe and permissible environment that enables and facilitates early locomotor training as well as training of many mobility-related skills. Compared to handheld assistive devices such as a walking frame, BWS systems provide specific benefits for locomotor trainings: 1) BWS maximizes controlled weight bearing on the legs which enhances lower limb electromyographic activity and interlimb coordination through appropriate sensory input and walking posture [4]. 2) BWS enables patients to swing their arms naturally, thus supporting forward motion and balance, the ability to control body posture dynamics that prevent falling [5], on a mechanical level [6–9]. 3) Natural arm swing induces rhythmic activation of the shoulder flexors and extensors which reinforces the patterned output of spinal locomotor networks, herewith supporting positive neuroplasticity [10–15]. 4) BWS prevents compensatory balance strategies that use the arms and thus results in maximized motor learning [16].

During the last years, BWS systems have become more elaborate and moved away from stationary, treadmill-coupled systems to highly transparent systems which allow both treadmill and overground walking. Transparent systems are defined by the ability to not apply any assistance/resistance to free motion [17]. This is especially relevant in evoking physiological gait patterns that allow seamless translation to a non-supported environment. Specifically, the attachment point must replicate the patient’s motion in sagittal and frontal planes with minimal delay. The basic construction principles behind BWS systems can be grouped into frame-based systems that are either stationary (e.g. Lokomat [18], G-EO [19], KineAssist MX [20]) or mobile (Biodex [21], Andago [22]). A second group of devices are ceiling mounted systems that are either based on a single rail mounting giving two degrees of freedom (2-DoF: Zero-G [23], Vector, Safe Gait) or multiple rail mounting providing three degrees of freedom (3-DoF: FLOAT [24], Rysen [25]). In contrast to frame-based systems and 2-DoF ceiling-mounted systems, 3-DoF BWS systems enable greater freedom of movement in all directions. The increased DoF in the most recent BWS systems allow physiological training of various activities with only minor deviations [3, 26–28] and might be an effective way to train balance control. Particularly for patients with neurological disorders, who fall twice as frequently as age-matched controls [29], such versatile balance training could improve functional recovery [30] and prevent fall-related loss of mobility and quality of life.

Increasing a BWS system’s DoF inherently increases the demands on patients’ balance capacity. If the challenge becomes too large, patients are prone to resort to crutches or walkers for external stabilization ultimately resulting in compensatory movement strategies. Preventing compensation and instead challenging balance control in a patient-tailored manner would be desirable [31]. An elegant solution which does not require reliance on external, arm-based support, would be a stabilizing mode as an integrated feature of the BWS system. Such a feature must be easily scalable to the functional level of the patient to provide a sufficiently large challenge for highly functioning patients, while avoiding overly challenging conditions for less functioning patients [32, 33].

A stabilizing feature of BWS systems could be especially helpful in the frontal plane. Maintenance of frontal plane balance during walking has been shown to require active control of lateral foot placement, which is highly dependent on successful integration of sensory feedback. In the sagittal plane, humans can use passive dynamic limb properties for stabilization. This reduces the relevance of active control of foot placement along the anteroposterior (AP) axis and therewith also the demands on the integration of sensory feedback [34]. One way of scaling the level of frontal plane support to each patient’s capabilities is to provide mediolateral (ML) stabilizing forces to the body in the frontal plane. Such ML forces can limit excessive center of mass (CoM) excursion in regard to the base of support (BoS), the minimum area enclosing the body’s contact with the ground [35], thus reducing balance demands. When describing BWS systems, we describe the position and forces acting on the end-effector which is considered as the attachment point of the BWS system to the BWS harness. Due to their architecture, single rail BWS systems inherently engender pendulum forces. When patients deviate too far from the midline, these forces move the BWS end-effector back into a position directly under the rail. While walking, patients can adapt their step width and cadence to use these forces for passive stabilization in the frontal plane [36–39]. Pendulum forces, however, cannot be adjusted to patients’ capacities or removed when patients become self-reliant with time. This limits their usefulness for adaptive stabilization and active balance training in the frontal plane. A different way to increase stability when walking in a straight line is by using lateral spring elements. This has been reported effective in young and elderly [40–43], as well as in patients with different central nervous system disorders [44, 45]. Employing lateral springs reduces energy and control costs, inferring that gait complexity and challenge may be decreased [40–42]. Compared to the pendulum forces of single rail systems, spring elements can be adjusted to produce larger or smaller stabilization forces. The spring-elements, however, are cumbersome - requiring additional hardware and attachment points - and are not location independent, so are only useful in a treadmill environment. A third option to influence gait stability are ML damping forces. These forces have the benefit that they can be adapted on the fly and can be applied independently of the user’s position and orientation in space. This will be especially valuable for 3-DoF overground BWS systems. However, damping forces oppose all movements along the defined axis, inferring that while unwanted COM displacements are reduced, desired displacements require increased effort. Hence, it remains to be investigated if damping of the BWS end-effector ultimately stabilizes ML COM motion and therewith proves beneficial for patients with balance impairments.

We investigated in this study how ML damping of the BWS end-effector affects frontal plane stability in able-bodied participants during walking. Stability during walking is commonly investigated using dynamic stability, the postural control process in which both the COM and the BoS are in motion [35]. It can be further divided into global or local dynamic stability, which are a system’s ability to resist large respectively small perturbations. In contrast to large external perturbations, small perturbations are naturally occurring fluctuations which arise from neuromotor noise or other internal perturbations [46]. In this study, we examined both global and local stability under transparent BWS and ML damped modes. This was compared to free walking. Our investigation aims at providing novel insight into how human-robot interaction during unloaded walking can subtly improve or challenge balance control without fundamentally distorting basic movement patterns. We hypothesized that mechanically damping the ML motion of the end-effector increases global and local frontal plane stability and therewith reduces balance demands. If this proves to be the case, ML damping can be applied in future studies with patients with balance impairments to test its effectiveness in a clinical setting.

## Methods

### Participants

Twenty-one young, able-bodied participants gave their informed written consent to participate in this study that was approved by the local ethics committee (KEK: 2016–01093) and conducted according to the Declaration of Helsinki and Good Clinical Practice. Inclusion criteria were that participants were older than 18 years of age and weighed less than 120 kg. The participants (11 female) were on average 26.8 ± 3.5 years old, 1.74 ± 0.1 m tall, weighed 65.7 ± 12.5 kg (mean ± 1 standard deviation, and walked on average with a self-selected velocity of 0.81 ± 0.14 m per second during the experiment).

### Experimental protocol

**Fig. 1:**
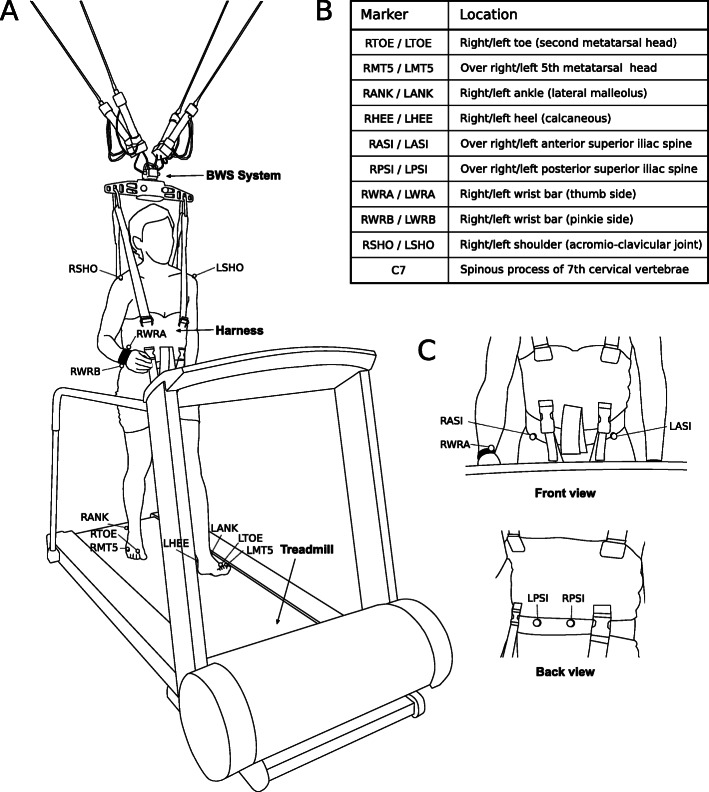
Experimental setup. **a** Subject walking with a harness and assistance from a 3-DoF, robotic BWS system (the FLOAT) on the treadmill. **b** List of used markers and their anatomical locations. C) Front and back view of pelvis marker placement next to BWS harness

Participants wore tight-fitting attire and were equipped with a specially modified BWS harness that allowed accurate placement of 19 spherical, retro-reflective markers with 14 mm diameter at bony landmarks (Fig. 1). Demographic information such as age, height, and weight were noted.

Afterwards, the participants walked for 5 min on a treadmill (Zebris FDM-T, Isny, Germany) wearing the harness but without BWS for familiarization purposes [47]. During this time, the participants were instructed to self-regulate their speed (increments of 0.028 m/s, no visual feedback) to achieve a “comfortable, everyday walking pace” which they would be able keep up for a total of 30–40 min. The median speed of minutes 3–5 was recorded as preferred gait velocity and was applied for the remaining experiment.

Following this familiarization, three conditions were applied in counterbalanced (equal distribution of the six possible orders among all subjects), pseudorandomized order: 30% BWS with damped ML motion (damped), 30% BWS without damping which enables fully transparent motion in the frontal plane (transparent) and typical walking without BWS but while wearing the harness (free).

In each experimental condition, participants walked for 10 min. The first 2 min were chosen to allow subjects to familiarize themselves with the condition. The remaining 8 min provided the necessary recording time for robust calculation of the parameters we used to quantify changes in stability [48, 49]. Between individual conditions, subjects were always detached from the BWS system and received a 5-min break during which they could rest in a standing position or lean on the treadmill’s handrails.

### Equipment

BWS was provided through the FLOAT, a cable-driven parallel robot, which allows large freedom of movement in an architecture-dependent workspace volume (2.35 m wide × 7.82 m long × 3.5 m high for our gait lab). Apart from safety (fall prevention) and up to 60% BWS, this active 3-DoF BWS system can provide damping along the AP or ML end-effector axes. We previously quantified the FLOAT’s transparency in a preceding experiment with a calibrated test rig that simulated walking of able-bodied participants and slow walking patients. These measurements showed that during transparent BWS mode only small interaction forces in the range of 3–18 N can be perceived in the horizontal plane during constant motion [50]. For the transparent and damped conditions, 30% BWS was applied. This reflects experience from clinical practice, where therapists frequently selected this level of assistance for early BWS training in patients that required substantial assistance. In the damped condition, ML stabilizing forces were rendered additionally to the 30% BWS. These were generated by continuously applying velocity-dependent damping to the ML end-effector axis:

$$ {F}_{damping}=-c\frac{dx}{dt}=- cv $$

c: viscous damping coefficient, v: mediolateral velocity of the BWS end-effector. The direction of the damping force was opposed to the end-effector mediolateral motion and a viscous damping coefficient of 120 N s/m (corresponding to the strongest damping setting available in the FLOAT) was used across all subjects with a maximal force magnitude saturated at 200 N. During conditions, marker positions were recorded at 200 Hz using 10 passive infrared cameras (T10/T20, Vicon, Oxford, UK). The FLOAT’s end-effector position was also recorded via optical motion tracking to ensure that the damping worked as intended.

### Outcome parameters

To investigate effects of BWS and damping on gait and dynamic stability, we selected 11 outcome parameters (Table 1). Two of these were spatiotemporal parameters along the AP axis: duty cycle and step length. These parameters were selected to verify that damping did not affect the spatiotemporal pattern along the AP direction. Five of these parameters quantified global dynamic stability along the ML axis: step width, ML COM sway, ML margins of stability (MoS), and the coefficients of variation (CoV) of step width and ML COM sway. ML MoS, which describes the positional relationship of COM and BOS at midstance, and the related measures step width and ML COM sway are direct measures of global dynamic stability. For the calculation of all COM-related parameters, we used an approximated COM (aCOM) calculated as the intersection of two vectors crossing the pelvis from left posterior spina iliac – right anterior spina iliac and left anterior spina iliac – right posterior spina iliac [51]. This was necessary because the BWS harness prevented reliable placement of upper trunk markers. Into the parameters describing global dynamic stability, we also included kinematic variability calculated from step width and ML aCOM sway. Many previous studies have shown that kinematic variability can be sensitive to changes in stability, e.g. between young and old [52] or fallers vs. non-fallers [53]. Changes in variability are however indirect measures of global dynamic stability and cannot provide causality because they are influenced by many other factors [54]. As descriptors of local dynamic stability, four nonlinear stability parameters based on the short-term maximum Lyapunov exponent (λ_s_) were included. The λ_s_ were calculated from ML and VT aCOM position (λ_s pos_) and velocity (λ_s vel_). λ_s_ takes the effect of the preceding step(s) on the next step into account while kinematic variability and direct measures of global dynamic stability treat each gait cycle as independent from others. λ_s_ is therefore especially suitable for detecting differences between position-timing dependent systems such as COM motion while walking [55–57]. λ_s_ can be used as a proxy measure of freedom in ML COM motion [58]. A higher λ_s_ indicates larger chaotic elements of a system, while λ_s_ close to zero indicates a stable system [59]. λ_s_ was chosen due to its validated representation of local dynamic stability which is not true for long-term Lyapunov exponents [60].

**Table 1:** Outcome parameters

Name	Definition	Unit	Primary effect axis
*AP spatiotemporal parameters*			
Duty cycle	Percentage of stance phase over the whole cycle duration	%	anteroposterior
Step length	Anteroposterior distance between contralateral heel markers at their respective heel strikes plus the distance the stance foot moved back with the treadmill belt during this time	m	anteroposterior
*Direct measures of global dynamic stability*			
Step width	Mediolateral distance between contralateral heel markers at their respective heel strikes	m	mediolateral
ML approximated COM (aCOM) sway	Difference between the ML extrema of the aCOM within each step	m	mediolateral
ML Margins of Stability (ML MoS)	Shortest distance of the floor-projected aCOM during midstance to the nearest edge of the base of support BoS was defined as the smallest convex hull spanning all points (heel, ankle, 5th metatarsal and 2nd metatarsal) in contact with the ground	m	mediolateral
*Indirect measures of global dynamic stability (kinematic variability)*			
Step width (CoV)	Standard deviation divided by the mean of step width	m	mediolateral
ML approximated COM (aCOM) sway (CoV)	Standard deviation divided by the mean of ML aCOM sway	m	mediolateral
*Measures of local dynamic stability (nonlinear parameters)*			
ML λ_s pos_	Short-term maximum Lyapunov exponent from time series of ML aCOM position	arbitrary unit	mediolateral
ML λ_s vel_	Short-term maximum Lyapunov exponent from time series of ML aCOM velocity	arbitrary unit	mediolateral
VT λ_s pos_	Short-term maximum Lyapunov exponent from time series of VT aCOM positions	arbitrary unit	vertical
VT λ_s vel_	Short-term maximum Lyapunov exponent from time series of VT aCOM velocity	arbitrary unit	vertical

### Data processing

For the calculation of the 11 selected parameters, the recorded motion data from minutes 2–10 of each condition was reconstructed (Nexus 2.7, Vicon, Oxford, UK) and labelled. From now on onwards, we term such processed, individual data sets “trials”. Custom MATLAB (R2018a, the MathWorks, Natick, USA) scripts were used for all further analysis. Gait events (heel strike, toe off) were algorithmically set for each trial based on velocity sign changes of the heel and toe markers according to Zeni et al. and visually verified [61]. For all parameters apart from λ_s_, marker data was segmented into gait cycles and linearly interpolated to 500 data points. Step length, step width, duty cycle, ML MoS, and ML aCOM sway were then extracted for each individual gait cycle and subsequently averaged per trial for each subject. The CoV for step width and ML aCOM sway was determined from the averaged gait cycle values and standard deviations obtained for each individual trial.

For the investigation of local dynamic stability using λ_s_, it is necessary to first reconstruct a higher dimension state-space from each dimension of the aCOM data [62]. For this, aCOM position and velocity traces in ML and VT directions were each downsampled to 50 Hz to remove contaminating artefacts. Trials were cropped to the same length, and the final 15 s of each trial were discarded to avoid transients (slowing down or stopping). We then reconstructed higher dimensional dynamics according to Takens’ Theorem [63] by embedding the original timeseries with time-delayed surrogate copies [62, 64, 65] as singularly defined by two parameters: a time delay (τ) and the number of embedding dimensions (D). These were determined separately for each trial: the minimal number of embedding dimensions was identified by observing the behavior of the closest geometrical neighbors using the false nearest neighbor method [66]. Similarly, the time delay between embedding dimensions was identified as the first local minima of the average mutual information function between the original time series and it’s lagged copy [67]. To enable comparison of λ_s_ between trials, we defined a common state space using the median number of embedding dimensions (D = 5) and median time delay (τ = 22 ms). The algorithm developed by Wolf was then used to determine λ_s_ for each trial [68].

### Statistical analysis

Statistical comparisons of all parameters were performed in SPSS (v25, IBM Corp., Armonk, USA). To validate if damping was effective, we first compared the ML and VT end-effector axes between transparent and damped conditions using a repeated measures t-test. Normality of data was tested with a Shapiro-Wilk test and the Holm–Bonferroni correction was used to counteract the problem of multiple comparisons. A one-way repeated measures multivariate analysis of variance (RM-MANOVA) was then used to assess if the three experimental conditions had significant different effects on the combination of the selected 11 outcome parameters: duty cycle, step width, step width variability, step length, aCOM sway, aCOM sway variability, MoS, ML λ_s pos_, ML λ_s vel_, VT λ_s pos_, and VT λ_s vel_. RM-MANOVA assumptions were checked beforehand including independence of observations, adequate sample size, absence of univariate and multivariate outliers, multivariate normality (using “MVN: a web-tool for assessing multivariate normality” [69]), linear relationship between each pair of dependent variables, and absence of multicollinearity. Pillai’s trace was selected as the test statistic of the RM-MANOVA due to its robustness against small violations of assumptions [70]. A significant RM-MANOVA was followed up with univariate, repeated measures analyses of variance for each outcome parameter to test which of the parameters were affected. Any violation of sphericity as detected by Mauchly’s test of sphericity was corrected using the Greenhouse-Geisser correction. Group differences were mapped to the individual conditions (free vs. damped vs. transparent) using pairwise multiple comparisons tests between all conditions corrected according to Bonferroni-Sidak. A significance level of alpha = 0.05 was set for all comparisons.

## Results

**Fig. 2:**
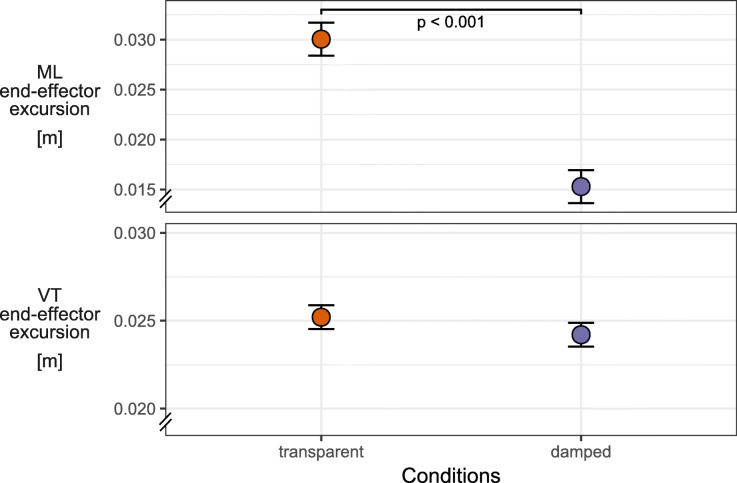
Effects of damping on BWS end-effector motion. Line plots of estimated marginal means over the transparent and damped conditions for mediolateral and vertical BWS end-effector excursion. Plot includes error bars specifying 95%-confidence intervals (within-subject, [71]) and statistically significant differences denoted by *p*-values. Abbreviations: ML – mediolateral, VT – vertical

The effectiveness of damping the ML end-effector axis was first verified by comparing the ML end-effector excursion during the damped against the transparent condition (Fig. 2). ML end-effector excursion was clearly reduced with damping (t (20) = 13.19, *p* < 0.001) while the VT excursion showed no significant difference (t (20) = 2.19, *p* = 0.081).

The RM-MANOVA showed a significant effect of the stability conditions on the measured parameters (V = 1.72, F (22, 62) = 17.04, *p* < 0.001). Separate univariate tests (individual analyses of variance) revealed that only the MoS were not affected by the differences between experimental conditions (Table 2).

**Table 2:** Repeated measures ANOVA test statistics for outcome parameters

Parameter	F-statistic	***P***-value
step length	F(1.41, 28.23) = 3.95)	*p* = 0.044
duty cycle	F(1.36, 27.21) = 37.88.15	*p* < 0.001
step width	F(1.45, 29.06) = 4.73	*p* = 0.014
ML aCOM sway	F(1.37, 27.46) = 65.93	*p* < 0.001
ML MoS	F(1.55, 31.00) = 1.59	*p* = 0.22
step width variability	F(1.41, 28.24) = 42.24	*p* < 0.001
aCoM sway variability	F(1.40, 28.04) = 49.81	*p* < 0.001
ML λ_s pos_	F(2, 40) = 27.58	*p* < 0.001
ML λ_s vel_	F(2, 40) = 56.86	*p* < 0.001
VT λ_s pos_	F(1.43, 28.53) = 34.14	*p* < 0.001
VT λ_s vel_	F(1.45, 29.03) = 41.86	*p* < 0.001

**Fig. 3:**
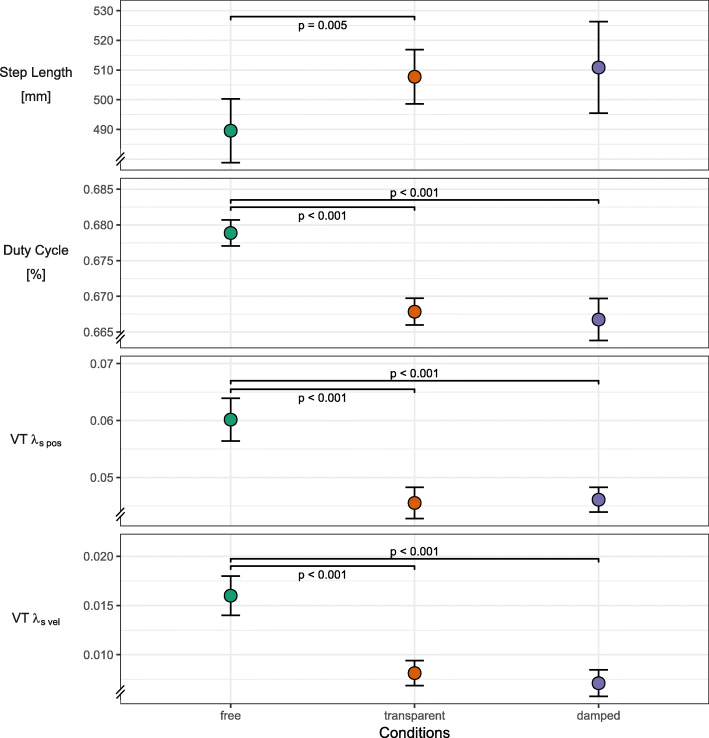
Effects of damped and undamped BWS on the anteroposterior and vertical axes. Line plots of estimated marginal means over the three stability conditions for outcomes related to the anteroposterior and vertical axes. Plot includes error bars specifying 95%-confidence intervals (within-subject, [71]) and statistically significant differences denoted by *p*-values. Abbreviations: VT – vertical, λ_s pos_ – short-term maximum Lyapunov exponent calculated from approximated center of mass position, λ_s vel_ – short-term maximum Lyapunov exponent calculated from approximated center of mass velocity

**Fig. 4:**
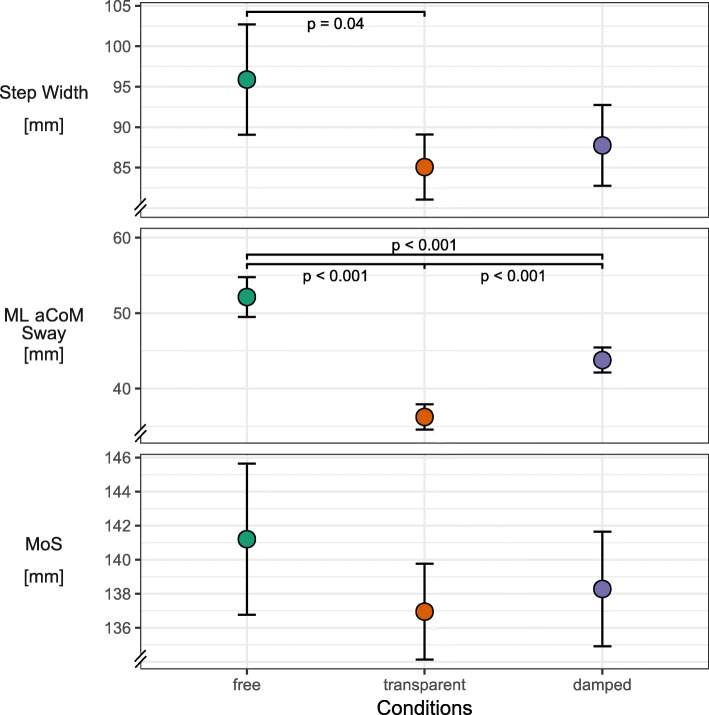
Effects of damped and undamped BWS on direct global dynamic stability. Line plots of estimated marginal means over the three stability conditions for outcomes reflecting direct global dynamic stability. Plot includes error bars specifying 95%-confidence intervals (within-subject, [71]) and statistically significant differences denoted by *p*-values. Abbreviations: ML – mediolateral, aCOM - approximated center of mass, MoS – Margins of stability

**Fig. 5:**
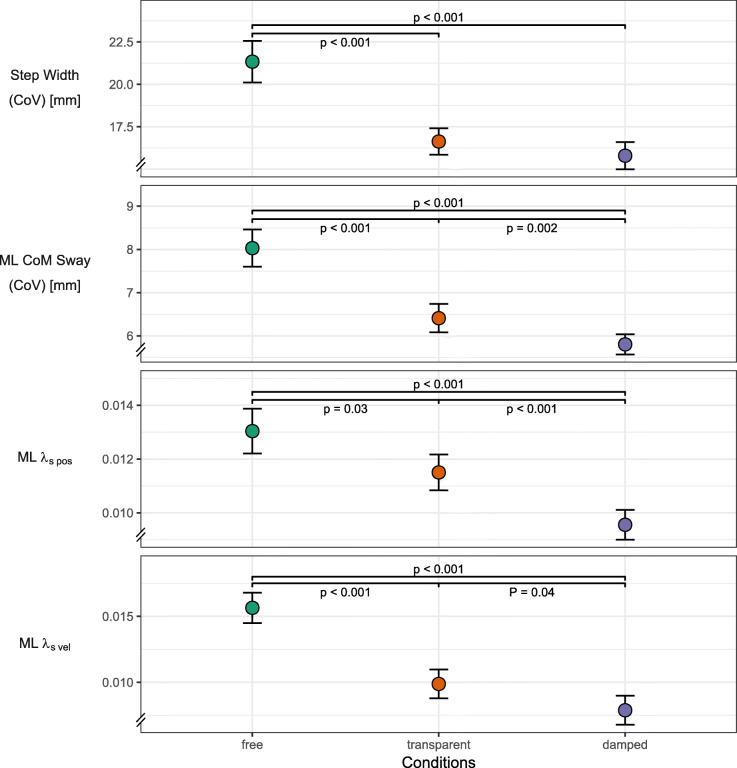
Effects of damped and undamped BWS on indirect global and local dynamic stability. Line plots of estimated marginal means over the three stability conditions for outcomes related to indirect global and local dynamic stability. Plot includes error bars specifying 95%-confidence intervals (within-subject, [71]) and statistically significant differences denoted by *p*-values. Abbreviations: ML – mediolateral, aCOM - approximated center of mass, CoV – coefficient of variance, λ_s pos_ – short-term maximum Lyapunov exponent calculated from approximated center of mass position, λ_s vel_ – short-term maximum Lyapunov exponent calculated from approximated center of mass velocity

Pairwise multiple comparisons for the statistically significant parameters allowed us to distinguish three parameter groups with different response patterns:

1. Spatiotemporal and local dynamic stability parameters along the VT axis showed differences only between the free condition and the transparent/damped conditions (Fig. 3). These parameters, whose response we allocated mainly to effects in the AP and VT axis, included duty cycle, step length and λ_s_ along the VT axis (λ_s pos_, λ_s vel_). The presence of BWS was the only separating experimental factor. While step length increased with the use of BWS, the remaining three parameters showed a clear decrease. Reduced VT λ_s pos_ and VT λ_s vel_ indicate increased local dynamic stability in the VT axis. A reduced duty cycle indicates a shift in relative duration from stance to swing.
2. For direct global dynamic stability parameters, the experimental conditions induced no coherent pattern and large variability was present for some of these parameters (Fig. 4). This included step width, ML aCOM sway, and MoS. ML aCOM sway was minimal during the transparent condition, increased in the damped condition, and further increased in the free condition. Step width was only decreased during the transparent condition and remained comparable between free and damped conditions. The MoS were not significantly affected by the experimental conditions.
3. Kinematic variability and local dynamic stability parameters along the ML axis showed a stepwise change from the free to transparent to damped condition (Fig. 5). This category included step width variability, ML aCOM sway variability, ML λ_s pos_, and ML λ_s vel_. All parameters showed a significant decrease from free to damped conditions. Between the transparent and damped conditions this decrease was less pronounced and was only not significant for step width variability.

## Discussion

In this study, we investigated the use of ML damping with a 3-DoF BWS system to support dynamic stability in able-bodied individuals walking on a treadmill. Participants walked in 3 different conditions: freely without BWS system attached, with 30% BWS but in a transparent device mode, and with 30% BWS and ML velocity-based damping. Our results show that damping indeed increases frontal plane dynamic stability compared to transparent and free walking conditions. This effect is mainly visible in kinematic variability and ML local dynamic parameters, while direct global dynamic stability parameters show no coherent response. Systematic changes of direct global dynamic stability parameters may be masked by compensatory movements aimed at accentuating active weight transfer. Adapting the damping level individually to the abilities of the participants in future studies might however reduce such compensatory effects.

### Body weight support effects

Literature on how BWS affects gait patterns of individuals both with [3, 27] and without neurological impairments [3, 26] indicates that small adaptations abound without grossly distorting either movement or myoelectric patterns. Our study results are in agreement with this. Additionally, we show that besides spatiotemporal parameters, dynamic stability is also clearly affected by BWS. From the selected outcome parameters, only the MoS were not decreased by BWS. Parameters which were equally affected in transparent and damped conditions were duty cycle, step length and local dynamic stability measured by λ_s_ along the VT axis. These parameters reacted primarily in response to the applied 30% BWS in both conditions. The decrease in duty cycle along with the subsequent increase in step length [27, 72, 73] has been frequently reported with regard to unloading. One explanation is that cadence decreases with BWS [26] which must be compensated by longer steps to maintain the same walking velocity [27] given by the fixed treadmill speed. With the reduced cadence, step duration becomes larger which is attributed to the influence of a prolonged swing phase while the stance phase remains less affected [27]. This change in relative swing and stance phase duration is reflected in the reduced duty cycle [21, 72–76]. An increase in local dynamic stability, as quantified by λ_s_ of the aCOM, has on the other hand not yet been reported in combination with body weight support conditions. Intuitively, it seems reasonable that unloading per se acts as a damper and reduces peak velocities, especially in earthward direction. This is reflected in the reduction of VT λ_s pos_ and VT λ_s vel_. BWS also reduces ML λ_s pos_ and ML λ_s vel_, indicating greater local dynamic stability in the frontal plane than during free walking. Variability of step width [58] along with ML aCOM sway and its variability were also reduced, which is in line with the general belief that BWS per se already increases dynamic stability [72, 77, 78]. Along the sagittal plane, Kyvelidou et al. found however increased λ_s_ and kinematic variability of the hip, knee, and ankle angles during walking with BWS on a treadmill [79]. They hypothesize that BWS might increase balance demands along the AP direction or that the instability is an effect of altered proprioception due to reduced limb loading. We did not measure dynamic stability based on lower limb joints, so there is the possibility that in both studies trunk stability increased while lower limb stability decreased. The relationship of the different contributors to stability is an important aspect to keep in mind when BWS is used to train patients with balance impairments and will require further investigation in the future.

There are other factors related to sensory perception which might contribute to the observed dynamic stability improvements. The linkage between harness and end-effector provides subjects with haptic feedback of their own position in relation to the end-effector position. This might aid subjects in sensing their own lateral position on the treadmill and may result in a less variable ML walking position [36]. Changes in global dynamic stability have been reported when subjects are allowed to lightly touch a side rail while walking [80, 81]. Light touch resulted in decreased ML COM sway and ML COM sway variability on a treadmill and ML MoS variability during overground walking, respectively. It is currently unclear if light touch would also affect local dynamic stability measured by λ_s_. Further studies are necessary to disentangle how much of the here observed dynamic stability gains can be attributed to BWS effects or stem from improved sensory perception.

### Effects of damping

To our best knowledge, our investigation of how ML damping of the end-effector affects dynamic stability during walking with BWS is completely novel. Our results indicate that adding ML damping on top of BWS provides enhanced dynamic stability. This is visible from reductions in global and local stability parameters between the free and transparent as well as between the transparent and damped conditions. Specifically, step width variability, ML aCOM sway variability and ML λ_s pos_ and λ_s vel_ were affected in a graded manner. Apart from step width variability for the transparent versus damped condition, all reductions were highly significant. Step width variability is often considered to reflect balance demands [34]. It increases for instance, when the eyes are closed [34, 54] or when visual perturbations are presented [82]. Multiple studies have previously investigated the sensitivity of step width variability to changed balance demands. These studies report increased variability in older adults [52, 83], decreased variability when walking with handrail usage [52], and a persistent decrease in variability with external stabilization through elastic springs [40–44]. Destabilizing force fields which increased the balance demands have been shown to increase step width variability [84]. Other studies have reported conflicting results and relate decreased step width variability to sensory impairments and fall risk [53]. Additionally, studies investigating attention during walking have also shown reduced step width variability under additional cognitive load [85]. The same was found for ML COM variability in a subsequent study and it was hypothesized that individuals adopt a more conservative gait pattern when attention is diverted from foot placement [86]. These studies would posit decreased variability of step width and ML COM as proxies of reduced stability. We therefore consider change in variability alone as not conclusive in differentiating between reduced or increased ML dynamic stability. λ_s_ on the other hand has proven to be more robust regarding the direction of change and can distinguish well between different stability demands [60, 87, 88]. As both kinematic variability and ML λ_s_ in our study show the same response, we consider this a robust indication that damping indeed improves dynamic stability in able-bodied participants.

Time-invariant parameters traditionally associated with global dynamic stability – such as step width [89], ML aCOM sway, and MoS [60, 90] – do however not seem to reflect the increased stability indicated by reduced variability and λ_s_ when damping is added. One potential explanation for this is that we did not perturb the system sufficiently. Variability and λ_s_ are thought to represent the system’s resilience to small perturbations as would be induced through the added ML damping. Global dynamic stability, on the other hand, represents the system’s resilience to large perturbations. These were potentially not encountered in our experimental conditions, explaining the lack of a cohesive response pattern in these parameters. A second potential explanation could be that the aCOM motion is exaggerated compared to the base of support to compensate for the ML damping. Exaggerated ML aCOM motion could help participants to retain a target, or “preferred”, step width. Veneman et al. have reported similar effects for half of their subjects when investigating pelvis fixations for a driven gait orthosis [91]. In our experiment, the BWS harness could well have restricted trunk excursion to some degree. Restricted trunk excursion has been reported to be linked with matched changes in step width [89, 92], giving indication that exaggerated trunk excursion could indeed help in maintaining a target step width. In summary, direct metrics of global dynamic stability show no coherent response to damping, presumably as the perturbation was insufficiently large or compensatory aCOM movement masked the response. The observed reductions in variability parameters and ML local dynamic stability coherently indicate an increase in stability when damping is applied.

### Clinical implications

From the current investigation, it is clear that damping provides a stabilizing effect in able-bodied individuals. The stabilization we showed here could be helpful for gait and balance training in patients with balance impairments. It is however still unclear how large this stabilizing effect is and if it provides enough stabilization to support patients with balance impairments. If the damping provides sufficient stabilization, training can be started at an earlier time point in rehabilitation when self-balance capacity is still limited. Using BWS in combination with a stabilization adapted to the patient’s needs, critical training parameters such as walking distance and training intensity can be increased. Both the early start of gait training and increase in training intensity has been shown to result in better recovery [93–95]. Finding the optimal balance for frontal plane stability assistance for each patient remains crucial, as overuse of stability support can result in patients slacking and becoming passive which decreases recovery [96]. An elegant aspect of using control-rendered damping is that the amount of stabilization can be tailored to each patient’s individual instantaneous level. This enables training at the patient’s optimal threshold while avoiding frequent and therefore time-consuming falls. The alternative option of adding handheld assistive devices to the training is in contradiction with established locomotor training principles [32, 97] as well as with emerging principles of reinforcing functional remapping through arm use [10, 11, 14].

From a handful of gait trainings of patients with incomplete spinal cord injury with a damped BWS end-effector, we can report that patients and their therapists subjectively perceive damping as an assistance which increases stability. A previous investigation by Wu et al. in which ML damping was provided without BWS directly to the pelvis of patients with incomplete spinal cord injury showed a reduction in step width and increased MoS [84]. This provides conceptual support that ML damping at the end-effector level can lead to improved dynamic stability in patients. Future investigations are needed to uncover the exact magnitude of stabilization that ML damping provides in clinical populations. In contrast to able-bodied subjects, psychological effects must be considered when measuring global and local stability in patients. In non-fallers, an increased fear of falling has been associated with higher λ_s_ [98]_._ In some groups of patients, dynamic stability could be increased alone through the perception of BWS as a safety aid. These and other design considerations are paramount when evaluating the approach of software-rendered viscous damping at the end-effector level as a stability aid for BWS locomotor training in clinical populations.

### Limitations

Three main limitations were identified in this study. First, COM kinematics were approximated through the intersection of the four pelvis markers. This has been reported to differ from gold standard COM calculations based on a full-body marker set especially along the ML axis under high walking speeds [99]. Under slower walking speeds comparable to ours, multiple studies have however shown that approximating the COM results in accurate COM estimations along the AP, ML, and VT directions [100–102]. The reduced marker set was chosen as even our reduced harness obscured trunk marker placements. Normal BWS harnesses make marker placements at both trunk and pelvis challenging, which renders the use of a full-body marker set for COM calculation questionable in this setting. Second, we described subjects’ motions through kinematics alone, which does not allow conclusions about how neuromuscular control was adapted in response to the external stabilization. Electromyography of the muscles involved in ML stability control along with modelling of the non-trivial human-robot system would be necessary to answer such questions which was beyond the scope of this study. Finally, it is important to keep in mind that the here obtained results can unfortunately not be generalized directly to overground walking. We decided to measure stability changes resulting from ML damping on a treadmill, as the nonlinear dynamic modeling approach requires a large amount of continuous walking data that cannot easily be recorded overground [103]. The necessary long measurement durations of around 10 min might also have led to learning or adaptation effects [84]. We counteracted this by introducing significant washout and familiarization periods before each measurement to limit carry-over effects to subsequent conditions.

## Conclusion

In this study, we provide first insights into the effect of ML damping in 3-DoF BWS systems on walking stability. We demonstrate that adding viscous damping during body weight supported treadmill walking increases local dynamic stability and attenuates movement variability in able-bodied subjects. This damped BWS mode can be used to support patients with balance impairments during locomotor training in a 3-DoF BWS system without introducing additional stability aids. This form of providing a stability aid is very elegant, as it allows easy, continuous adjustment of the damping to each patient’s instantaneous capacity. In able-bodied subjects, we however also observed what we believe to be compensatory aCOM movements to counteract movement limitations of the end-effector when the system was damped. This underlines the importance of tailoring the amount of support to each patient’s capacity to achieve an optimum between stability support and challenge during training. Further studies are necessary to show how patient groups react to different levels of mediolateral damping of the BWS end-effector in terms of walking stability.

## Data Availability

The datasets used and/or analyzed during the current study are available from the corresponding author on reasonable request.

## Abbreviations

λ_s_: Short-term maximum Lyapunov exponent
aCOM: Approximated center of mass
AP: Anteroposterior
BWS: Body weight support
BoS: Base of support
COM: Center of mass
DoF: Degrees of freedom
ML: Mediolateral
MoS: Margins of stability
RM-MANOVA: Repeated measures multivariate analysis of variance
VT: Vertical
